# Ending Neglect of Older People in the Response to Humanitarian Emergencies

**DOI:** 10.1371/journal.pmed.1001357

**Published:** 2012-12-18

**Authors:** Unni Karunakara, Frances Stevenson

**Affiliations:** 1 Médecins Sans Frontières (MSF), Geneva, Switzerland; 2 Mailman School of Public Health, Columbia University, New York, United States of America; 3 HelpAge International, London, United Kingdom

## Abstract

Unni Karunakara and Frances Stevenson argue that humanitarian policy and practice must adapt to ensure that the needs of older people in humanitarian emergencies are addressed.

**Summary Points**

- Older people make up a significant and growing number of those affected by humanitarian crises, yet they are often not sought out or prioritised within the humanitarian response.
- Humanitarian agencies, donors, and international bodies neglect older people's health and nutrition.
- The gaps in knowledge and research about the needs of older people in emergencies are considerable.
- Older people are not monitored in emergencies and they are not prioritised despite evidence of disproportionate mortality and morbidity in this group.
- We call for policy changes by humanitarian agencies and donors to ensure that the needs of this vulnerable group are met.

## Introduction

At the heart of humanitarian action are the principles of humanity and impartiality. All people have equal value and dignity; the exclusion of an individual or a group on the grounds of nationality, religion, or politics is contrary to the humanitarian ethos [1]. We suggest that neglect on the grounds of age should be added to this list. But first, what do we mean by “older”? Variation in life expectancy between countries makes it difficult to agree upon the age at which someone becomes old. The United Nations (UN) definition is 60 years and older, but in developing countries, where most humanitarian crises occur, 50 years is an appropriate cut-off and the one we use in this article [2].

Emergency aid has failed to address the needs of older people in emergencies [3]. High death rates among older populations in crises are common, partly due to their inherent vulnerability and partly due to services that inadequately deal with their needs often because these are not well identified or understood [3]–[6]. For example, an assessment by Médecins Sans Frontières (MSF) of how it met the needs of older people in emergencies in Haiti and South Sudan showed that after initial registration of individuals at health clinics, disaggregated data beyond the under-5 and over-5 year age groups were not collated [7]. Issues with mobility and vision that increase in prevalence with age can mean that older people are more at risk in crises and are less able to access care and support [8],[9]. Vulnerable groups such as older people are therefore likely to bear a disproportionate share of health problems [10]. Thus, specific interventions are often necessary to prevent these groups from suffering disproportionately.

Here we describe the neglect of older people's health and nutrition needs by humanitarian agencies, donors, and international bodies. Our aim is to stimulate thinking and action on how older people are treated in humanitarian emergencies.

## Ageing Population

Global changes in conflicts and demographic changes mean that older people constitute a growing number of those affected by humanitarian crises [11]. The population of older people in developing countries is growing faster than in developed countries [12]. By 2050, more than 80% of older people will live in developing countries, compared to the 60% who do so today, where disasters are more likely to occur and their effects are greater [13]. In some areas, particularly remote rural areas, high HIV prevalence, conflict, and economic migration have led to high proportions of older people [14].

Older people are less likely to flee in times of conflict due to hardships associated with travel and reluctance to leave home, land, and possessions [15],[16]. They face constraints to returning home after long periods of displacement [17]. Many are not able to travel to health facilities, stand in queues for food distributions, carry heavy packages of food or containers of water, or compete with younger people for relief supplies [18]. And, contrary to a common assumption, older people are often not cared for within their families. Following the 2010 floods in Pakistan, around 10% of the older population was living without family support [19]. In camps for internally displaced people in Darfur, half the older people live alone [20].

## How Are Older People Neglected?

### In Monitoring and Surveillance

Effective humanitarian assistance relies on assessment and surveillance data. Almost all guidance, such as the Sphere Handbook that defines minimum standards for humanitarian assistance [21], requires collection of sex- and age-disaggregated data. In practice, most adult data are aggregated, leaving the profile and needs of older groups invisible [22]. MSF did an exploratory analysis of 2012 data from the South Sudan refugee crisis. During a 6-week period, data were disaggregated by age into under 5, 5–50, and >50-year-olds. The mortality rate in the oldest group was over four times that of the 5- to 50-year-olds and over twice that of the under 5 s, during a time when the crude mortality rate was just below the emergency threshold (one death per 10,000 population per day) (Philipp du Cros, personal communication).

Similarly, the nutritional status of children under 5 years old, assessed by measuring MUAC (mid-upper arm circumference), is the standard indicator of whether a nutrition intervention is needed in the general population. However, few nutrition surveys are carried out among older adults [18]. HelpAge International did a nutrition survey in the refugee camps of Dadaab, Kenya, with the aim of influencing standard practice. It showed that older people were vulnerable to malnutrition, although they had not been included in other nutritional surveys or in supplementary or therapeutic nutrition programmes [23].

### In Guidelines for Chronic and Communicable Disease

Older people have specific health risks and needs. Global ageing is recognised as a major driver of non-communicable disease in developing countries where older people are at especially high risk of cardiovascular disease, strokes, diabetes, and dementia [24]. Yet non-communicable chronic disease is neglected in humanitarian responses, and there are virtually no guidelines for the management of chronic medical conditions after disasters [25]. The latest revision of the Sphere Handbook, however, recognises the increasing evidence of acute complications from chronic diseases in disasters and encourages their treatment [21].

Older people may also be at increased risk from communicable diseases [26],[27]. Yet older people are rarely identified as an at-risk group for communicable diseases or their specific needs considered by infection control programmes [28].

### By Donors

Institutional donors do not treat humanitarian assistance for older people as a priority. Although in 2010 12.5% of the world's population was older than 60 years, analyses of humanitarian projects funded through the UN Consolidated Appeals Process revealed that just 0.3% included any activity that specifically targeted the needs of older people, while in 21 countries there were no projects targeting older people in the past 2 years [29],[30].

### By Humanitarian Agencies

Aside from research by specialist agencies such as HelpAge International, there is little evidence of humanitarian organisations considering how best to meet the needs of older people in emergencies. A World Health Organization (WHO) review of the needs of older people in emergencies noted that humanitarian agencies “do not assess older people's needs, nor . . . address their needs” [31]. The MSF study highlighted a lack of specific organisational policies on meeting the needs of older people even though they were viewed as a vulnerable group. MSF's response to their needs was variable and unexpectedly better in the emergency setting of Haiti than in the slow-onset emergency of South Sudan [7]. MSF is in the process of translating these research findings into operational policies and practice.

## Next Steps

From analysis of the literature, reviews of MSF programmes and policies, and discussion within our organisations, we have identified priority areas for action and research for humanitarian agencies, research institutes, international bodies, and donors (Box 1). As the numbers of older people affected by humanitarian crises and disasters increase, humanitarian actors need to adapt policy and practice to ensure that the needs of older people are consistently and continually considered and that this vulnerable group is no longer neglected.

**Box 1. Priority Areas for Including Older People in Humanitarian Agendas**

**Practice and policy**

- Collecting age-disaggregated data on who is accessing humanitarian assistance to ensure that older people are not being excluded.
- Ensuring all surveillance, needs, and vulnerability assessment data are disaggregated for older age groups.
- Systematically screening older people in nutrition surveys using MUAC.
- Providing adequate care in emergencies for chronic diseases and conditions, including palliative care and pain relief.
- Ensuring that infection control programmes take into account the differing presentation and needs of older people.

**Research**

- Researching the optimum nutritional support for older people in emergency settings.
- Conducting operational research into the best way to ensure that older people's health needs are comprehensively met in humanitarian programmes.
- Conducting operational research to determine how best to improve older people's access to humanitarian assistance both in open settings and in camps.

**Funding**

- Institutional funding bodies should ensure that the needs of older people are included in project proposals where appropriate and funded.

## Abbreviations

MSF: Médecins Sans Frontières
MUAC: mid-upper arm circumference
UN: United Nations
WHO: World Health Organization

## References

[1] Médecins Sans Frontières. MSF Charter. Available: http://www.msf.org/msf/articles/2011/03/the-medecins-sans-frontieres-charter.cfm. Accessed 17 July 2012.

[2] Definition of an older or elderly person: Proposed Working Definition of an Older Person in Africa for the MDS Project. Geneva: WHO. Available: http://www.who.int/healthinfo/survey/ageingdefnolder/en/index.html. Accessed 2 October 2012.

[3] ADA, Irish Aid, Age Action Ireland (2012) Ageing & development: a guide for development organisations. Available: http://www.wheel.ie/sites/default/files/Age%20Action%20Ageing%20and%20Development%20Guide-1.pdf. Accessed 2 October 2012.

[4] Assefa F, Jabarkhil M, Salama P, Spiegel P (2001) Malnutrition and mortality in Kohistan District, Afghanistan, April 2001. JAMA 286: 2723–2728. 11730450 10.1001/jama.286.21.2723

[5] Spiegel PB, Salama P (2000) War and mortality in Kosovo, 1998–99: an epidemiological testimony. Lancet 355: 2204–2209. 10881894 10.1016/S0140-6736(00)02404-1

[6] Salama P, Assefa F, Talley L, Spiegel P, Van Der Veen A, et al (2001) Malnutrition, measles, mortality, and the humanitarian response during a famine in Ethiopia. JAMA 286: 563–571. 11476658 10.1001/jama.286.5.563

[7] Médecins Sans Frontières (2012) Older people in crisis: a review of MSFs approach to vulnerability and needs. London: Médecins Sans Frontières. Available: http://www.msf.org.uk/older_people_in_crisis_2012. Accessed 21 October 2012.

[8] HelpAge International, IFRC (2011) Guidance on including older people in emergency shelter programmes. Available: http://www.helpage.org/what-we-do/emergencies/guidance-on-including-older-people-in-emergency-shelter-programmes/. Accessed 15 October 2012.

[9] HelpAge International, IDMC (2012) The neglected generation: the impact of displacement on older people. Available: http://www.helpage.org/what-we-do/emergencies/the-neglected-generation-the-impact-of-displacement-on-older-people/. Accessed 15 October 2012.

[10] Mannan H, Amin M, MacLachlan M, The EquitAble Consortium (2011) The EquiFrame manual. Dublin: The Global Health Press. p. 5.

[11] Spiegel P, Checchi F, Colombo S, Paik E (2010) Health-care needs of people affected by conflict: future trends and changing frameworks. Lancet 375: 9341–9345. 10.1016/S0140-6736(09)61873-0 20109961

[12] Shrestha LB (2000) Population aging in developing countries. Health Aff 19: 204–212. 10.1377/hlthaff.19.3.204 10812800

[13] Population Division of the Department of Economic and Social Affairs of the United Nations Secretariat (2010) World population prospects: the 2010 revision. Available: http://esa.un.org/unpd/wpp/. Accessed 17 July 2012.

[14] Samuels F, Wells J (2009) The loss of the middle ground: the impact of crises and HIV and AIDS on ‘skipped-generation’ households. Overseas Development Institute (ODI): Project Briefing: 33, November 2009. Available: http://www.odi.org.uk/resources/docs/5062.pdf. Accessed 17 July 2012.

[15] Karunakara UK (2004) The demography of forced migration: displacement and fertility in the West Nile region of northern Uganda and southern Sudan. Johns Hopkins University. DrPH.

[16] Harrell-Bond BE (1986) Imposing aid: emergency assistance to refugees. Oxford: Oxford University Press.

[17] Wells J (2012) The neglected generation: the impact of displacement on older people. HelpAge International/Internal Displacement Monitoring Centre. Available: http://www.helpage.org/download/4fdf5078382b5. Accessed 17 July 2012.

[18] Wells J (2005) Protecting and assisting older people in emergencies. Humanitarian Practice Network. London: Overseas Development Institute (ODI).

[19] HelpAge International (2011) Analysis of older people's ongoing vulnerabilities in Sindh, Pakistan. HelpAge International. Available: http://www.helpage.org/download/4fdf5078382b5/. Accessed 2 October 2012.

[20] HelpAge International (2008) Older people in Africa: a forgotten generation. HelpAge International. Available http://eng.zivot90.cz/uploads/document/205.pdf. Accessed 17 July 2012.

[21] The Sphere Project (2011) The Sphere handbook: humanitarian charter and minimum standards in humanitarian response. Belmont Press. 10.1111/j.0361-3666.2004.00245.x 20958782

[22] Mazurana D, Benelli P, Gupta H, Walker P (2011) Sex and age matter: improving humanitarian response in emergencies. Feinstein International Center, Tufts University; August 2011.

[23] Fritsch P, Myatt M (2011) Nutrition and baseline survey of older people in three refugee camps in Dadaab. HelpAge International. October 2011. Available: data.unhcr.org/horn-of-africa/download.php?id = 729. Accessed 2 October 2012.

[24] UN General Assembly (2010) UN General Assembly 65th session. High-level meeting of the General Assembly on the prevention and control of non-communicable diseases. Note by the Secretary-General; 13 September 2010.

[25] Chan EY, Sondorp E (2008) Including chronic disease care in emergency responses. Humanitarian Exchange Magazine 41 December 2008.

[26] Gavazzi G, Herrmann F, Krause KH (2004) Aging and infectious diseases in the developing world. Clin Infect Dis 39: 83–91. 15206058 10.1086/421559

[27] Kwok J, Swarthout T, Fritsch P, Raza A, Newport M (2012) Loving the older people in times of cholera: preliminary findings from a study to analyse care and outcomes for cholera patients treated by Médecins Sans Frontières Operational Centre Amsterdam in Haiti and Zimbabwe 2008–12. Poster presented at MSF Scientific Day 2012, London. Available: http://issuu.com/msfuk/docs/pdf/1. Accessed 17 July 2012.

[28] Chappuis F, Alirol E, Worku DT, Mueller Y (2011) High mortality among older patients treated with pentavalent antimonials for visceral leishmaniasis in East Africa and rationale for switch to liposomal amphotericin B. Antimicrob Agents Chemother 55: 455–456. 21078947 10.1128/AAC.01298-10 PMC3019666

[29] HelpAge International (2012) A study of humanitarian financing for older people and people with disabilities, 2010–2011. HelpAge International, 2012. Available: http://www.helpage.org/download/4f4222be3ce76. Accessed 17 July 2012.

[30] HelpAge International (2010) A study of humanitarian financing for older people. Available: www.helpage.org/download/4cd984edb1147/. Accessed 17 July 2012.

[31] Hutton D (2008) Older people in emergencies: considerations for action and policy development. WHO. pp. 33–34.

